# Preclinical efficacy assessment of umbilical cord mesenchymal stem cell therapy for acute liver failure

**DOI:** 10.22038/ijbms.2025.88181.19046

**Published:** 2025

**Authors:** Danpeng Shen, Fan Xie, Weikang Chen, Shixian Lv, Shengxian Chen, Xi Qin, Sha Zhu

**Affiliations:** 1 Department of Immunology, School of Basic Medical Sciences, Zhengzhou University, Zhengzhou 450001, Henan, China; 2 Hangzhou Zhiyuan Research Institute, Zhouhang 310020, Zhejiang, China; 3 Zhengzhou Mycelial Protein Biotechnology Co., Ltd, Zhengzhou, 450001, Henan, China

**Keywords:** Acute liver failure, Immunomodulation Inflammation, Neutrophils, Rats, Umbilical cord-derived- mesenchymal stem cells

## Abstract

**Objective(s):**

Acute liver failure (ALF) is a life-threatening condition marked by rapid hepatocellular damage. This study investigates the therapeutic efficacy of human umbilical cord-derived mesenchymal stem cells (hUCMSCs) in a rat model of ALF.

**Materials and Methods:**

ALF was induced using D-galactosamine and lipopolysaccharide in rats. hUCMSCs were administered intravenously at different time points and dosages. Liver function, inflammatory cytokine levels, histopathology, neutrophil infiltration, and survival rates were evaluated. Additionally, biodistribution was tracked using 89Zr-labeled hUCMSCs, and fresh versus cryopreserved cells were compared.

**Results:**

Administration of hUCMSCs, especially at the prog-2h time point, significantly improved survival and liver histology. Treatment reduced ALT and AST levels and modulated pro- and anti-inflammatory cytokines. Neutrophil infiltration was alleviated by both fresh and cryo-preserved hUCMSCs. Biodistribution data revealed hepatic enrichment of hUCMSCs peaking at 24 hr post-injection.

**Conclusion:**

hUCMSCs exhibit strong immunomodulatory and hepatoprotective properties in ALF, offering promise for clinical translation. Timing and dosage significantly influence therapeutic efficacy, and cryopreserved cells maintain functionality comparable to that of fresh cells.

## Introduction

ALF refers to severe liver function failure caused by acute liver damage due to various reasons, characterized by progressive liver cell necrosis, reduction or loss of liver function, and reduced or absent coagulation function, often leading to multiple organ dysfunction syndrome and death. ALF is an acute critical illness with the characteristics of rapid onset, rapid progression, severe illness, and poor prognosis, with a mortality rate as high as 70% to 90%. Patients with ALF often have significantly enlarged and hardened livers, with a sharp liver margin and tenderness. Liver function is significantly impaired, mainly manifested as jaundice, ascites, bile stasis, hepatic encephalopathy, etc. At the same time, patients with ALF often exhibit multiple organ dysfunction syndrome (MODS), including multiple systems such as the nervous system, kidneys, lungs, cardiovascular system, etc., such as consciousness disorders, respiratory distress, hypotension, arrhythmia, etc. In addition, ALF patients often have severe coagulation abnormalities and complications such as gastrointestinal bleeding (1-3).

The incidence of ALF in China is relatively low, but due to the large population, the number of deaths caused by ALF is still high. ALF is becoming increasingly common in younger age groups, particularly between 20 and 40 years old, with a peak incidence between 20 and 30 years old (4). The leading causes of ALF in China are drug-induced liver injury and hepatitis B virus infection. Other causes include viral hepatitis, drug overdose, autoimmune liver disease, Wilson’s disease, and so on. The Asia-Pacific region is home to more than half of the global population and accounted for 62.6% of global deaths due to liver diseases in 2015 (5). The mortality rate of ALF is high, particularly for ALF caused by viral infections, which can reach 60%-80%. Liver transplantation is currently the primary treatment for ALF, but due to the shortage of donors, many patients cannot receive liver transplantation in time. Therefore, it is necessary to actively explore other treatment methods, such as maintenance therapy and artificial liver support systems (6, 7). 

hUCMSCs mainly intervene in the treatment of ALF by promoting liver cell proliferation and differentiation, releasing growth factors and cytokines, suppressing inflammatory reactions, and regulating immune responses. hUCMSCs can differentiate into various types of cells, including liver cells. After injection, stem cells can settle in liver tissues and differentiate into liver cells, promoting liver cell proliferation and differentiation, and improving liver function. hUCMSCs can secrete multiple growth factors and cytokines, which can promote liver cell proliferation and differentiation, suppress inflammatory reactions, and promote tissue repair and regeneration. ALF is usually associated with inflammatory reactions, and hUCMSCs can promote treatment by suppressing inflammatory reactions. hUCMSCs can secrete various anti-inflammatory cytokines, including IL-10, HGF, PGE2, and TGF-β (8), which inhibit inflammatory reactions and reduce inflammatory damage (8). hUCMSCs can also inhibit the activity and proliferation of immune cells, such as T cells, B cells, and granulocytes, thereby reducing immune reactions and suppressing liver cell apoptosis during the acute phase of ALF (9, 10).

The combined application of D-GAL (D-galactosamine) and LPS (lipopolysaccharide) is a standard method for establishing an ALF rat model, which can be used to study liver diseases related to liver dysfunction, hepatocyte necrosis, and inflammation, among other conditions. D-GAL is a polysaccharide naturally found in certain plants and can cause rapid liver damage in rats through injection or gavage. LPS is a type of lipid found in bacterial cell walls that can induce inflammation in rats through injection (11, 12). The combined use of D-GAL and LPS can enhance their effects on the liver, leading to more severe liver damage and inflammation (13, 14). 

In this experiment, the ALF rat model was established by combining D-GAL and LPS. Pre-clinical efficacy exploration was performed by administering hUCMSCs via tail vein injection before and after modeling, as well as injecting different doses of hUCMSCs after modeling. Efficacy evaluation was conducted by assessing survival time, liver histological structure, secretion of inflammatory factors, and liver function (alanine aminotransferase (ALT), aspartate aminotransferase (AST), total bilirubin (TBIL), alkaline phosphatase (ALP), and albumin (ALB)). The study further examines the pharmacokinetics and biodistribution of ^89^Zr-labeled hUCMSCs and compares the anti-inflammatory and regenerative capabilities of fresh versus cryopreserved cells. These findings aim to optimize hUCMSCs therapy for ALF and provide a solid foundation for translating preclinical insights into clinical practice.

## Materials and Methods

### Experimental design and animals

Wistar clean-grade female rats weighing 180-200 g (160 in total) were purchased from Zhejiang Vital River Laboratory Animal Technology Co., Ltd. and approved by the Institutional Animal Ethics and Use Committee of Zhengzhou University (ZZUIRB2024-217). The rats were purchased in two batches and raised in a sterile environment. The experimental rats were randomly assigned using a random number table before the start of the animal experiment.

**Figure 1:**
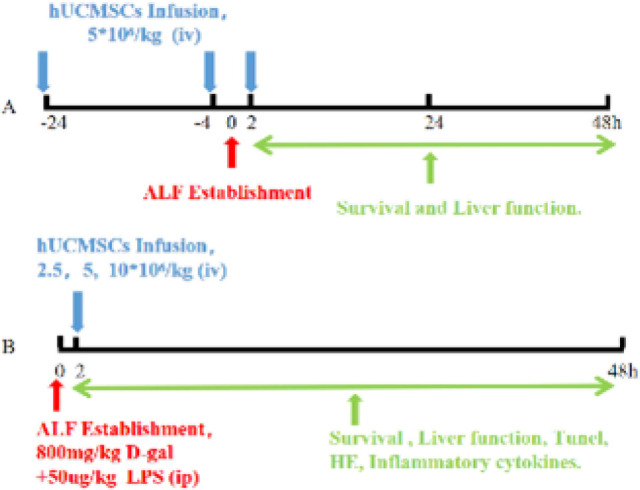
Flowchart of hUCMSCs treatment for ALF

The first batch of 70 rats was used to explore the therapeutic effects of different administration time points on the ALF model. The experimental groups included: normal group (n=5), pre-24h group, pre-4h group, prog-2h group (n=15)(15). Each group of rats was injected with hUCMSCs at a dose of 5×10^6^/kg, and the normal group was given the same dose of PBS. The ALF rats were induced by intraperitoneal injection of 800 mg/kg and 50 ug/kg D-gal LPS mixture (11, 14), and the survival time and liver function were detected within 48 hr of each group at the dying state (Figure 1A).

The second batch of rats consisted of 70 rats, and different doses of hUCMSCs were injected via the tail vein two hours after modeling (16). The model establishment method was the same as before. The experimental groups consisted of the following: normal group (n=5), model group, low-dose group (2.5×10^6^/kg), middle-dose group (5×10^6^/kg), and high-dose group (10×10^6^/kg)(n=15). The normal group and model group were given the same dosage of PBS. The status of each rat group was recorded, and the liver and blood of dying rats were collected. The survival time, liver function, liver pathology, necrosis status, and inflammatory factors (TNF-α, IL-6, IL-10, IFN-γ, IL-18, TGF-β, and HGF) in the liver and blood of rats in the dying state were detected (Figure 1B).

Due to the high mortality rate among ALF rats, a pharmacokinetic distribution study of hUCMSCs was conducted using nine healthy rats in this experiment. Three rats served as the control group and received a single intravenous injection of ^89^Zr-oxalate, while six rats constituted the experimental group and received a single intravenous injection of 4×10^6^ ^89^Zr-oxalate-labeled hUCMSCs. Micro-PET/CT scans were performed at six time points: 1, 24, 72, 168, 240, and 336 hr post-injection to record the distribution of hUCMSCs in the rat liver (17).

Additionally, we compared the ability of fresh and cryopreserved hUCMSCs to inhibit neutrophils in ALF rats (18, 19). After modeling, a total of 1×10^7^ cells were injected immediately, with four rats in the model group, fresh group, and cryo-preserved group, and three rats in the normal group. The rats were sacrificed 16 hr later, and their livers were collected to assess the proportion of neutrophils.

### Preparation of hUCMSCs

The hUCMSCs used in this experiment were sourced from Shanghai Quansheng Biotechnology Co., Ltd. They were isolated and extracted from human umbilical cords (obtained with ethical approval from Dongguan Chang’an Hospital)(2109507150102035) using enzymatic digestion and expanded to the sixth generation in a GMP workshop before being cryo-preserved. The purity of the cells was determined by flow cytometry using surface markers (CD14, CD19, CD31, CD34, CD45, CD73, CD90, CD105, and HLA-DR-Phycoerythrin (PE)). Adipogenic differentiation of hUCMSCs was analyzed using Oil Red O staining, chondrogenic differentiation was explored using Alcian blue staining, and osteogenic differentiation was analyzed using Alizarin Red staining (20).

### Immunomodulatory function detection of hUCMSCs

Before conducting animal pharmacodynamics experiments with hUCMSCs, we verified their immunomodulatory function *in vitro* using cell-based assays (21). In the experiment assessing the inhibition of TNF-α secretion by hUCMSCs, we established three experimental groups: Peripheral Blood Mononuclear Cells (PBMCs)+Phytohemagglutinin (PHA), hUCMSCs+PHA, and PBMC+hUCMSCs+PHA. hUCMSCs were digested and plated in a six-well plate (3×10^5^ cells/well), and after overnight attachment, the supernatant was discarded. PBMCs were added at a 3×10^6^ ratio, and PHA was added to each group at a final concentration of 10 ng/mL. After co-culturing for 3 days, the supernatant was collected, and TNF-α levels were measured using an ELISA kit (Biolegend).

In the experiment investigating the effect of hUCMSCs on the proportion of T-reg cells in PBMCs, we set up two experimental groups: PBMC and PBMC+MSC. hUCMSCs were digested and plated in a six-well plate (3×10^5^ cells/well), and after overnight attachment, the supernatant was discarded. PBMCs were added at a 3×10^6^ ratio, and the cells were co-cultured for 3 days. Afterward, the floating cells were collected, and the proportion of T-reg cells was analyzed by flow cytometry. Surface staining was performed using CD4/CD25 antibodies (BD), followed by intracellular staining with Foxp3 antibodies (BD).

### Survival analysis

In the time-point and dose-response experiments, after establishing the model, the survival time of rats was recorded within 48 hr, and the survival status of the rats was observed (22). When rats were dying, their blood and liver were collected and stored for later detection.

### Liver function analysis

Collect blood and liver samples from each group of rats within 48 hr, and take 200 μl of serum to perform liver function analysis using an automatic biochemical analyzer (HITACHI). The detected indicators include: ALT, AST, TBIL, ALB, and ALP. Some dead rats were not sampled promptly during the experiment, resulting in missing data in each experimental group.

### Measurement of inflammatory cytokines in blood and liver tissues

Collected blood and liver samples from each group of rats within 48 hr. Centrifuged the blood at 10,000 g for 15 min to obtain serum, which should be aliquoted and stored at -80 ^°^C. Took an equal amount of the left lateral lobe of the liver, freeze-thaw it repeatedly, grind it, and then centrifuge at 1500g for 15 min. Collected the supernatant and measure the protein concentration before storing it at -80 ^°^C. Used the LEGEND plex Multifactor Assay Kit (Biolegend) to detect the levels of inflammatory factors (TNF-α, IL-6, IL-18, IFN-γ, IL-10, and HGF) in both serum and liver tissue fluid. Used the TGF-β1 ELISA KIT (Biolegend) to measure the level of TGF-β.

### Pathological analysis

Took the left lateral lobe of the liver from each group of rats, fixed, embedded it in paraffin, and then sliced it. Stained the slices with hematoxylin-eosin (HE) to observe the pathological state of the liver structure.

### Tunel analysis

Took the left lateral lobe of the liver from each group of rats, fixed, embedded it in paraffin, and then sliced it. Cell apoptosis in the liver was measured using the 1-step TUNEL Apoptosis Assay Kit (Beyotime) according to the manufacturer’s instructions.

### Distribution analysis

^89^Zr-labeled hUCMSCs were administered to each rat via tail vein injection at a dose of 4×10^6^ cells (n=6)(17). Micro-PET/CT scans were performed at 1 hr, 24 hr, 72 hr, 168 hr, 240 hr, and 336 hr after injection. The Micro-PET/CT scan data were processed using Pmod software, and the standard uptake value (SUV) of the liver was calculated.

### Neutrophil detection in the liver

Sixteen hours after intravenous injection of hUCMSCs into ALF rats, the model group, Cryo-hUCMSCs group, and Fresh-hUCMSCs Group rats were anesthetized. Their livers were collected, followed by washing in PBS. A small portion of the left lobe tissue was excised, washed, and cut into small pieces for grinding. After grinding, the tissue was filtered through a 70 µm filter and treated with red blood cell lysis buffer. The cells were then stained with antibodies against CD11B and Ly6G. Double-positive cells for CD11B and Ly6G were identified as neutrophils (18).

### Data analysis

The GraphPad Prism 8 software was used for statistical analysis. Data are expressed as means±standard deviation (SD). The log-rank test was used to compare survival rates among the four groups, and statistical analysis was carried out using one-way analysis of variance. *P*-values less than 0.05 were considered to be statistically significant.

## Results

### Detection of phenotype and trilineage differentiation ability of hUCMSCs.

**Figure 2:**
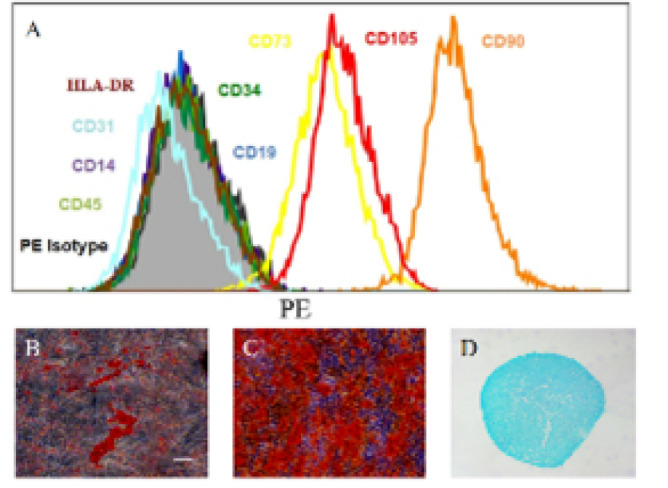
Detection of surface markers and trilineage differentiation ability of hUCMSCs

Surface marker analysis of hUCMSCs using flow cytometry revealed negative expression of CD14, CD19, CD31, CD34, CD45, and HLA-DR, while revealing positive expression of CD73, CD90, and CD105 (Figure 2A). Additionally, the differentiation potential of hUCMSCs was evaluated, and the results showed successful transdifferentiation into adipocytes (analyzed by Oil Red O staining) and osteoblasts (analyzed by Alizarin Red)(Figures 2B-D).

### Immunomodulatory Functions of hUCMSCs

**Figure 3:**
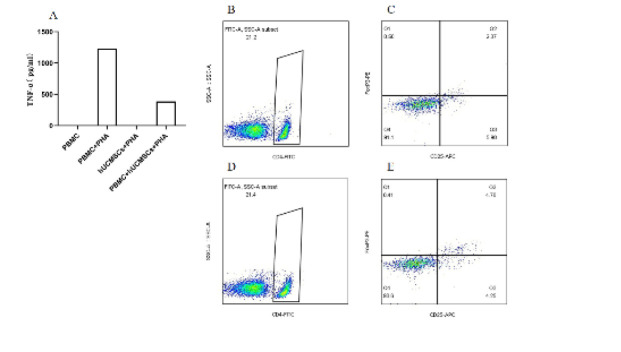
hUCMSCs inhibit the secretion of TNF-α and increase the proportion of T-regulatory cells in PBMC

PHA effectively stimulates the activation of PBMC. After three days of stimulation, the TNF-α content in the culture supernatant significantly increases. However, when PBMCs are co-cultured with hUCMSCs, a noticeable reduction in TNF-α levels is observed, as hUCMSCs themselves do not secrete TNF-α (Figure 3A). After three days of culture without PHA, the proportion of T-regulatory cells in PBMC is 0.50% (Figures 3B-C). However, after co-culturing with hUCMSCs for three days, the proportion of T-regulatory cells increases to 1.01% (Figures 3D-E), doubling the initial proportion (20).

### The effect of hUCMSCs on the survival time and liver function of ALF rats

**Figure 4:**
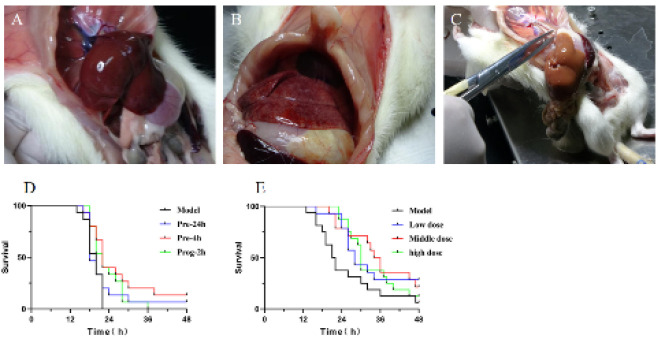
Typical images of liver surface morphology in normal/ALF rats and survival curves

Rats injected intraperitoneally with D-gal and LPS maintained normal activity and responsiveness within 12 hr. Some rats exhibited features such as mental depression, poor responsiveness, drowsiness, and yellow urine after 12 hr of injection. In the liver tissue of ALF rats, the liver surface exhibited scattered or patchy congestion, a tense capsule, and a lack of luster. In severe cases, there was extensive necrosis (Figure 4B) and jaundice (Figure 4C). This was significantly different from the red-brown color, soft texture, and glossy appearance of the normal rat liver (Figure 4A).

After 14 hr, ALF rats began to die one after another, and this continued until 48 r had passed. At the same time, combined with previous model exploration results, it was found that the rats developed strong tolerance after surviving for 48 hr and could continue to survive, with liver function indicators gradually returning to normal. In the intervention experiment at different time points, pre-24h and pre-4h, as well as prog-2h, could prolong the survival time of ALF rats (Figure 4D). Moreover, there were significant statistical differences between the groups with pre-4h and prog-2h and the model group (*P*≤0.01, *P*≤0.05). In the dose-effect relationship experiment, different doses of hUCMSCs effectively prolonged the survival time of rats, and there were significant statistical differences between each treatment group and the model group (*P*≤0.05). However, a dose-dependent relationship was not observed (Figure 4E).

### The effect of hUCMSCs on the liver function of ALF rats

**Figure 5:**
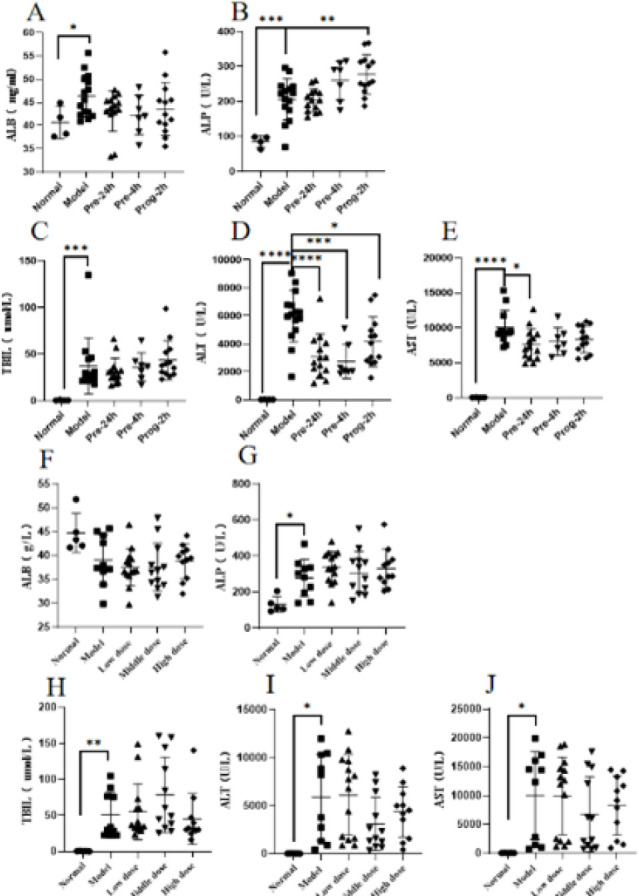
Detection of liver function indicators in normal/ALF rats

In the experimental study, at different time points of drug administration, ALB, a marker of chronic liver disease, did not show any significant differences (Figure 5A). Compared to the normal group, both the model group and treatment group showed a significant increase in ALP. As the time of drug administration was delayed, the treatment group showed a significant increase, with the Prog-2h group showing substantial differences (*P*≤0.01)(Figure 5B). Compared to the normal group, the model group showed a significant increase in TBIL, ALT, and AST (*P*≤0.05, *P*≤0.0001, *P*≤0.0001), whereas the treatment group did not exhibit inhibition of TBIL secretion but effectively inhibited the secretion of ALT and AST. Pre-24h Group, Pre-4h Group, and Prog-2h Group showed statistically significant differences in inhibiting ALT secretion (*P*≤0.0001, *P*≤0.001, *P*≤0.05), and only Pre-24h Group showed statistically significant differences in inhibiting AST secretion (*P*≤0.05) (Figures 5C-E).

In the dose-response experiment, the results for ALB and ALP were consistent with those of the time point exploration experiment (Figures 5F and G). Compared to the normal group, the model group showed a significant increase in TBIL, ALT, and AST (*P*≤0.01, *P*≤0.05, and *P*≤0.05). Compared to the model group, the treatment group did not exhibit inhibition of TBIL secretion. However, the Middle dose Group and High dose Group showed a trend of inhibiting the secretion of ALT and AST, while the Low dose Group did not show any significant differences (Figures 5H-J).

### Effect of hUCMSCs on the secretion of inflammatory factors in the blood and liver of ALF rats

**Figure 6:**
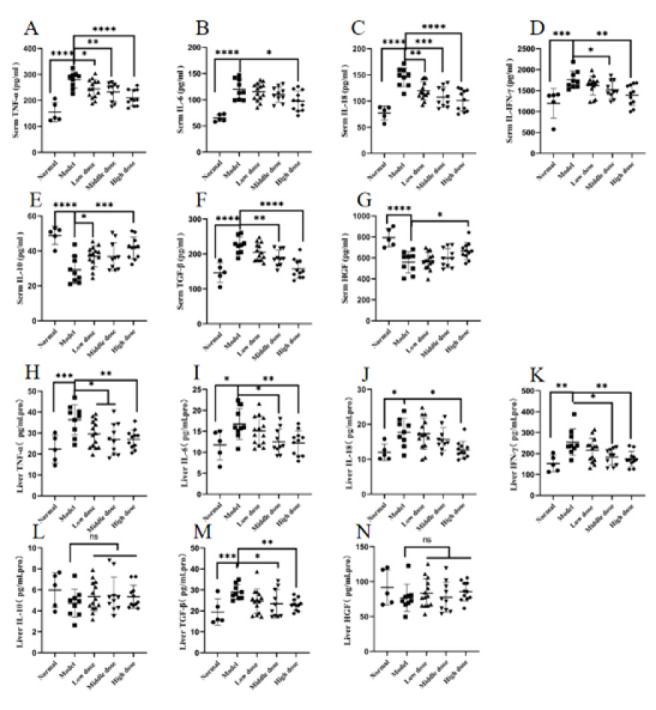
Detection of inflammatory factors in the blood and liver of normal and ALF rats

This study investigated the anti-inflammatory and alleviating effects of hUCMSCs on ALF by measuring the secretion of inflammatory factors in rat serum and liver. Compared with the normal group, the levels of inflammatory factors (TNF-α, IL-6, IL-18, and IFN-γ) in the serum and liver were significantly increased in the model group, with almost all showing statistically significant differences (Figures 6A-D, H-K). Compared with the normal group, the secretion of IL-10 in serum was significantly decreased in the model group (*P*≤0.0001); compared with the model group, the secretion of IL-10 in all treatment groups increased, and the Low dose Group and High dose Group showed significant statistical differences (*P*≤0.05 and *P*≤0.001)(Figure 6E), while there was no change in the secretion of IL-10 in the liver (Figure 6L). Compared with the normal group, the levels of TGF-β in serum and liver were significantly increased in the model group, with statistical differences (*P*≤0.0001 and *P*≤0.001); compared with the model group, the levels of TGF-β in serum and liver showed a decreasing trend in all treatment groups, with the Middle dose Group and High dose Group showing significant statistical differences (*P*≤0.01, *P*≤0.0001; *P*≤0.05, and *P*≤0.01) (Figure 6F, M). Compared with the normal group, the secretion of HGF in serum was significantly decreased in the model group (*P*≤0.0001); compared with the model group, the secretion of HGF increased in all treatment groups, and the High dose Group showed significant statistical differences (*P*≤0.05) (Figure 6G), while there was no change in the secretion of HGF in the liver (Figure 6N).

### Effect of hUCMSCs on the pathological structure and cellular necrosis of the liver in ALF rats

**Figure 7:**
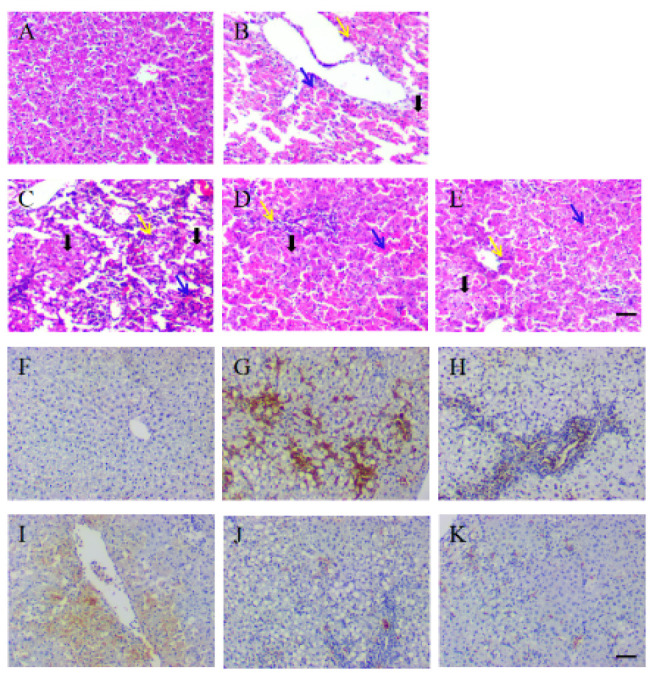
Pathological and TUNEL detection images of the liver in normal and ALF rats

Through HE staining of rat liver left lateral lobe tissue, pathological structural analysis showed that the morphology and boundaries of the normal group were normal (Figure 7A). The model group exhibited a disrupted structure, characterized by widespread hepatocyte necrosis and inflammatory cell infiltration (Figure 7B). The treatment group still exhibited hepatocyte necrosis and inflammatory cell infiltration; however, the disease symptoms were relatively mild, and some rat livers retained intact and clear structures (Figures 7C-E).

Analysis of cell death was performed on the left lateral lobe of rat liver tissue using Tunel staining. The results showed that the liver in the normal group was normal, with no signs of apoptosis or necrosis (Figure 7F). The model group (with rapid disease progression) and the low-dose group had more cell death (Figures 7G, I), while some rats in the model group, middle-dose group, and high-dose group with slower disease progression showed better self-healing or curative effects, with no extensive liver cell death (Figures 7H, J-K).

### Research on the distribution of hUCMSCs in the rat liver

**Figure 8:**
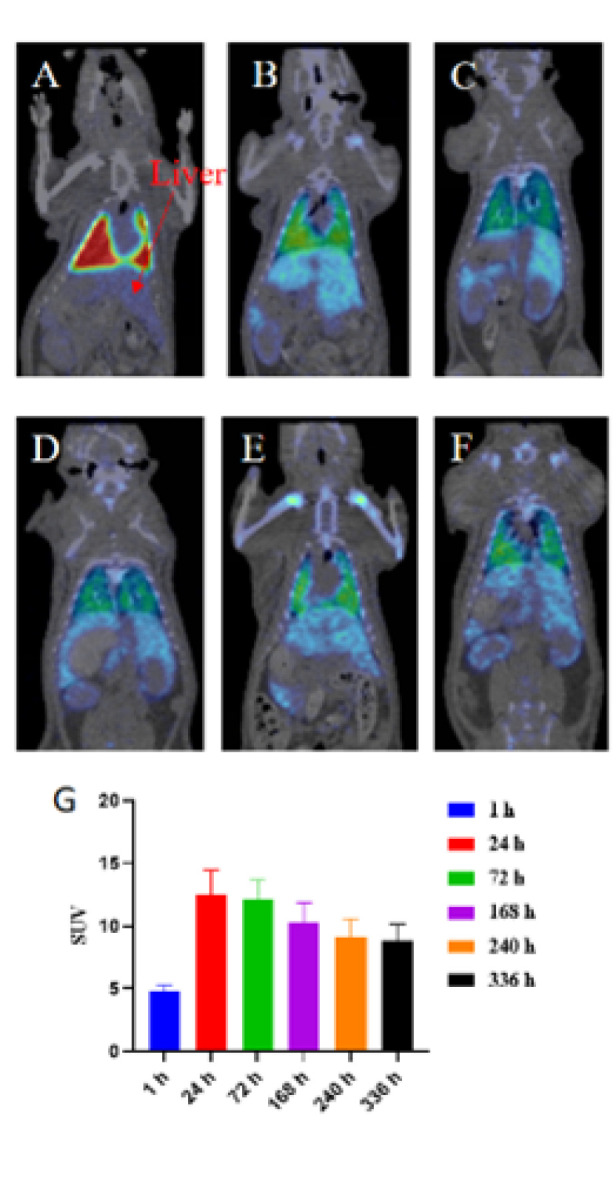
Distribution of ^89^Zr-labeled hUCMSCs in rat liver

^89^Zr-labeled hUCMSCs were injected into normal rats through the tail vein at a dose of 4×10^6^ cells, and liver distribution was studied. Micro-PET/CT scans were performed at six different time points: 1 hr, 24 hr, 72 hr, 168 hr, 240 hr, and 336 hr. The results showed that the SUV value in the liver was the lowest at one hour after injection, and the SUV signal value of the liver in rats reached its maximum at 24 hr after injection and then began to decrease, which continued until 336 hr (Figures 8A-G).

### Effect of cryo-hUCMSCs and fresh-hUCMSCs on the proportion of neutrophils in the liver of rats with ALF

**Figure 9:**
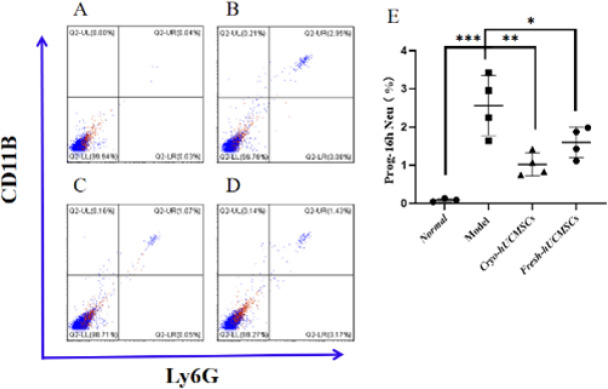
Percentage of neutrophils in the liver of ALF rats 16 hr after intravenous injection of hUCMSCs

The proportion of neutrophils in the rat liver was determined by flow cytometry, which identified CD11B- and Ly6G-positive cells as neutrophils. The results showed that neutrophils were nearly absent in the liver of rats in the normal group (Figure 9A). Compared to the normal group, the model group exhibited a significant increase in neutrophils in the liver (*P*≤0.001)(Figure 9B). However, compared to the model group, Cryo-hUCMSCs and Fresh-hUCMSCs significantly reduced the proportion of neutrophils in the livers of ALF rats (*P*≤0.01 and *P*≤0.05) (Figures 9C-E).

## Discussion

In this study, hUCMSCs demonstrated robust immunomodulatory functions and therapeutic efficacy in an ALF rat (22, 23). The observed benefits included prolonged survival time, reduced secretion of inflammatory cytokines, decreased neutrophil infiltration into the liver, lowered liver enzyme levels (ALT and AST), and mitigated widespread hepatocyte necrosis and apoptosis (24). Furthermore, pharmacokinetic analysis using ^89^Zr-labeled hUCMSCs revealed significant liver homing, with the highest cell accumulation observed 24 hr after intravenous administration.

The immunomodulatory capabilities of hUCMSCs are well-documented, particularly their ability to suppress the activation and proliferation of T cells, NK cells, and macrophages, as well as the secretion of inflammatory cytokines (8, 25). However, fewer studies have examined their effects on neutrophil activity within the ALF liver. Neutrophils are closely associated with liver damage and disease progression in ALF, where excessive neutrophil recruitment to the liver triggers hyperimmune activation and a cytokine storm, exacerbating hepatocyte damage and necrosis, ultimately leading to multi-organ injury. In our study, hUCMSCs effectively reduced neutrophil recruitment and activation by secreting anti-inflammatory cytokines, such as IL-10 and TGF-β (26), thereby alleviating neutrophil-mediated liver damage. Both fresh and cryopreserved hUCMSCs significantly reduced neutrophil infiltration into the liver of ALF rats (27, 28). This experiment preliminarily demonstrates that the current cryo-preservation process for hUCMSCs is stable, with consistent functionality in acute applications. However, aspects such as secretory factors, metabolic activity, immunomodulatory functions, and potential repair capabilities remain key areas of concern and will be the focus of our future research efforts (29, 30). However, this study lacked a thorough dose-dependent analysis, which warrants further investigation at higher dosages. Given the known effects of hUCMSCs on macrophage polarization in vitro, future studies will explore the roles of type I and type II macrophages in liver pathology.

The pharmacokinetic analysis, based on ^89^Zr-labeled hUCMSCs, showed that intravenously administered hUCMSCs peaked in the liver at 24 hr (17). This finding prompted us to evaluate the effects of administering hUCMSCs 24 hr prior to ALF induction. Surprisingly, different timing regimens did not significantly affect survival outcomes, consistent with previous findings in similar studies (15). We hypothesize that during the acute phase of ALF, the homed hUCMSCs may lack the efficiency needed to counteract systemic hyperinflammation via extracirculatory mechanisms, limiting their impact on survival. Conversely, early intervention during the initial stages of ALF could yield a more favorable therapeutic response by mitigating inflammation across multiple organ systems. Unexpectedly, we also observed fatty liver or jaundice in surviving rats. However, pathological images of fatty liver were not included in this study; whether fatty liver protects against acute hepatic necrosis and apoptosis will be a key focus in future research.

Different dose groups were designed based on clinical trial protocols to explore their therapeutic effects. While an increasing trend in efficacy was observed with higher doses—evident in inflammation suppression, improved survival, and alleviated liver damage (26)—no statistically significant differences were detected. It is possible that the highest dose in this study did not reach the pharmacodynamic threshold. Additionally, we observed a dramatic surge in AST and ALT levels in the blood of moribund rats, distinct from those in rats with normal activity. This phenomenon provides valuable insight for clinical biomarker evaluation. Unlike chronic liver disease, where ALB and ALP levels tend to increase, these markers did not show significant changes in acute liver failure. hUCMSCs did not significantly inhibit the secretion of TBIL, which might suggest limitations in affecting bilirubin metabolism. This observation could be linked to the complex mechanisms of bilirubin clearance and secretion in the ALF model, such as impaired bile excretion pathways. Future research could employ transcriptomics or metabolomics to understand better the influence of hUCMSCs on key pathways involved in bilirubin metabolism (31).

This study provides experimental evidence to guide future research on hUCMSCs for treating acute-on-chronic liver failure (ACLF) and lays the groundwork for clinical applications (32). Numerous clinical trials and preclinical pharmacological studies have validated the therapeutic efficacy of hUCMSCs (33). Patients receiving hUCMSCs therapy exhibited improved liver function markers, such as decreased ALT and AST levels, as well as elevated albumin levels. Imaging and histological assessments confirmed reduced liver inflammation and enhanced hepatic regeneration. Furthermore, survival rates and clinical outcomes were markedly improved in the hUCMSCs-treated groups compared to the control groups, with fewer complications and faster recovery times (22, 34). These clinical results highlight the robust therapeutic potential of hUCMSCs in restoring liver function and promoting recovery in patients with ALF.

In this study, the disease model demonstrated rapid progression in the experimental animals, driven by multiple factors, including extensive hepatocyte necrosis and apoptosis, cytokine storms, coagulation dysfunction, and sepsis-induced multi-organ failure. Our observations primarily focused on the impact of hUCMSCs on hepatocyte function and liver inflammation, providing only a preliminary understanding. Although we considered evaluating prothrombin time (PT) and activated partial thromboplastin time (APTT), the blood samples were insufficient for additional tests after liver function and inflammatory factor analyses were completed. Additionally, during the experimental process, we noted rapid blood coagulation in moribund rats, which warrants further investigation in future studies.

While hUCMSCs demonstrated anti-inflammatory and tissue repair effects in this model, their therapeutic efficacy for such acute conditions remains limited. To address this, we plan to employ an ACLF animal model to further validate the pharmacological effects of hUCMSCs. We also aim to conduct more extensive dose-dependent studies and investigate specific signaling pathways to deepen our understanding of their mechanisms.

A: Evaluation of therapeutic efficacy after tail vein injection of hUCMSCs at different time points before and after ALF model establishment. B: Evaluation of therapeutic efficacy after tail vein injection of different doses of hUCMSCs after ALF model establishment. The blue arrow indicates the time of hUCMSCs injection, the red arrow indicates the time of ALF model establishment, and the green arrow indicates the detection index

hUCMSCs: Human umbilical cord-derived mesenchymal stem cells, ALF: Acute liver failure

A: Surface markers CD14, CD19, CD31, CD34, CD45, and HLA-DR are negatively expressed, while CD73, CD90, and CD105 are positively expressed in hUCMSCs. B: Oil red O staining was performed to analyze adipogenic differentiation of hUCMSCs. C: Alcian blue staining was performed to explore chondrogenic differentiation of hUCMSCs. D: Alizarin red staining was performed to analyze osteogenic differentiation of hUCMSCs. The scale bar is 50 μm in (B, C) and 200 μm in (D) hUCMSCs: Human umbilical cord-derived mesenchymal stem cells; PE: Phycoerythrin

A: Concentration of TNF-α in the supernatant after co-culturing PBMCs with hUCMSCs under PHA stimulation for 3 days. B-C: Proportion of T-reg cells (CD4+CD25+FOXp3+) after culturing PBMCs for 3 days. D-E: Proportion of T-reg cells (CD4+CD25+FOXp3+) after co-culturing PBMCs with hUCMSCs for three days

hUCMSCs: Human umbilical cord-derived mesenchymal stem cells; PBMCs: Peripheral blood mononuclear cells

A: Normal rat liver morphology. B: Liver morphology of ALF rats with rapid disease progression and short survival time. C: Liver morphology of ALF rats with slow disease progression and long survival time. D: Survival curves of rats after tail vein injection of hUCMSCs at different time points before and after ALF model establishment. E: Survival curves of rats after tail vein injection of different doses of hUCMSCs after ALF model establishment

hUCMSCs: Human umbilical cord-derived mesenchymal stem cells, ALF: Acute liver failure

A-E: Detection of liver function (ALB, ALP, TBIL, ALT, and AST) in rats after tail vein injection of hUCMSCs at different time points before and after ALF model establishment. The Normal Group: n=4, the other groups: n=15. However, some samples were severely hemolyzed and could not be detected, so they were excluded from the analysis. One sample was excluded from the Pre-24h Group, eight samples were excluded from the Pre-4h Group, and two samples were excluded from the Prog-2h Group. F-J: Detection of liver function (ALB, ALP, TBIL, ALT, and AST) in rats after tail vein injection of different doses of hUCMSCs after ALF model establishment. The Normal Group: n=5, the other groups: n=15. However, some samples were severely hemolyzed and could not be detected, so they were excluded from the analysis. Five samples were excluded from the Model Group, 1 sample was excluded from the Low dose Group, three samples were excluded from the Middle dose Group, and four samples were excluded from the High dose Group. Data are presented as mean±SD; *P≤0.05, **P≤0.01, ***P≤0.001, and ****P≤0.0001, vs Model

hUCMSCs: Human umbilical cord-derived mesenchymal stem cells, ALP: Alkaline phosphatase, ALB: Albumin, ALT: Alanine aminotransferase, AST: Aspartate aminotransferase, TBIL: Total bilirubin, ALF: Acute liver failure

A-G: Secretion levels of TNF-α, IL-6, IL-18, IFN-γ, IL-10, TGF-β, and HGF in the blood of rats. H-N: Secretion levels of TNF-α, IL-6, IL-18, IFN-γ, IL-10, TGF-β, and HGF in the liver of rats. The Normal Group: n=5, the other groups: n=15. However, some samples had severe hemolysis and could not be detected, so they were excluded. The Model Group excluded 6, the Low Dose Group excluded 1, the Middle Dose Group excluded 5, and the High Dose Group excluded 4. Data are presented as mean±SD; *P≤0.05, **P≤0.01, ***P≤0.001, and ****P≤0.0001, vs Model

ALF: Acute liver failure

A-E: HE staining pathological structure images of the Normal Group, Model Group, Low dose Group, Middle dose Group, and High dose Group, respectively. The blue arrow indicates the bleeding site; the yellow arrow indicates inflammatory cells; the black arrow indicates the necrotic area. F-K: TUNEL staining pathological structure images of the Normal Group, Model Group (rapid disease progression), Model Group (slow disease progression), Low dose Group, Middle dose Group, and High dose Group, respectively. The red area indicates the necrotic cells, and the magnification of the staining is 100 times

ALF: Acute liver failure

A-F: Micro-PET/CT scanning images at six time points (1 hr, 24 hr, 72 hr, 168 hr, 240 hr, and 336 hr) after intravenous injection of ^89^Zr-labeled hUCMSCs in rats. G: SUV values in rats at each time point, n=6. Data are presented as mean±SD

hUCMSCs: Human umbilical cord-derived mesenchymal stem cells

A-D: Flow cytometry images of neutrophils in the liver (left lateral lobe) of the Normal Group, Model Group, Cryo-hUCMSCs Group, and Fresh-hUCMSCs Group, respectively. E: Percentage of neutrophils in the liver of rats in different groups. Normal Group: n=3, other groups: n=4. Prog-16h indicates injection of hUCMSCs 16 hr after establishing the ALF model. Data are presented as mean±SD; *P≤0.05, **P≤0.01, and ***P≤0.001, vs Model. Figures A-D use overlay scatter plots, where red dots represent the isotype control group and blue dots represent the experimental group. Data is presented through this overlay format

hUCMSCs: Human umbilical cord-derived mesenchymal stem cells, ALF: Acute liver failure

## Conclusion

In conclusion, this study highlights the potential of hUCMSCs in treating ALF, although their ability to elicit rapid therapeutic responses in acute conditions remains uncertain. hUCMSCs could serve as an adjunctive therapy during the disease’s active phase or as an intervention in early or recovery stages, with promising therapeutic outcomes. Moreover, genetic modification of hUCMSCs to enhance their efficacy is a promising avenue for future research, as is the exploration of therapeutic targets. Finally, a more detailed comparison of the functional and paracrine differences between fresh and cryo-preserved hUCMSCs will further clarify their therapeutic potential in ALF.

## Data Availability

All data and materials generated or used during the study are available from the corresponding authors upon reasonable request.

## Declaration

OpenAI’s ChatGPT (GPT-4) was used to refine grammar, clarify sentence structure, and ensure consistency in scientific writing. No AI tools were used for data generation, statistical analysis, or image production.
